# Cohort profile: ORICAMs, a French cohort of medical workers exposed to low-dose ionizing radiation

**DOI:** 10.1371/journal.pone.0286910

**Published:** 2023-06-08

**Authors:** Julie Lopes, Clémence Baudin, Juliette Feuardent, Hervé Roy, Sylvaine Caër-Lorho, Klervi Leuraud, Marie-Odile Bernier

**Affiliations:** 1 Laboratory of Epidemiology (LEPID), Institute for Radiological Protection and Nuclear Safety (IRSN), Fontenay-aux-Roses, France; 2 Office for the Analysis and Monitoring of Occupational Exposure (BASEP), Institute for Radiological Protection and Nuclear Safety (IRSN), Fontenay-aux-Roses, France; Universiti Teknologi Malaysia, MALAYSIA

## Abstract

Medical personnel represent the largest group of workers occupationally exposed to ionizing radiation. Although the health risks associated with occupational exposure to low doses of ionizing radiation in the medical field have been investigated in several national cohorts, no study has been conducted in France to date. The ORICAMs (Occupational Radiation Induced Cancer in Medical staff) cohort is a nationwide French longitudinal cohort of medical workers exposed to ionizing radiation aiming to investigate the risk of radiation-associated cancer and non-cancer mortality. The ORICAMs cohort was set up in 2011 and includes all medical personnel monitored for ionizing radiation exposure with at least one dosimetric record in the SISERI database (the national registry for monitoring ionizing radiation exposure in workers) over the period 2002–2012. Causes of death were abstracted from death certificates and coded according to ICD-10. The follow-up ended on 31/12/2013. Standardized mortality ratios (SMRs) were calculated by cause of death to compare the mortality in the cohort to that in the French population, by gender, age group and calendar period. Among the 164,015 workers included in the cohort (60% women) a total of 1,358 deaths (892 in male and 466 in female) were reported. The observed number of all-cause deaths was significantly lower than expected based on national rates in both male (SMR = 0.35; 95% CI: 0.33, 0.38; n_deaths_ = 892) and female (SMR = 0.41; 95% CI: 0.38, 0.45; n_deaths_ = 466). This analysis leads to the conclusion that mortality in French workers exposed to medical radiation is significantly lower than the national reference rates. However, these results based on a comparative analysis with national rates may be impacted by the healthy worker effect towards low SMRs, and do not enable to establish a potential relationship between occupational exposure and mortality risk, even if we may suspect an impact of high SES of these professionals on the observed decreased mortality. Thus, further dose-response analyses based on individual ionizing radiation exposure and job’s type will be conducted to characterize correlation between risk of cancer mortality and occupational exposure.

## Introduction

Ionizing radiation has been used in the medical field for over a century and has become a key component of diagnostic and therapeutic medical practices in today’s modern societies [1]. Through medical discoveries in the use of radiation and radioactive materials, technological advances in diagnostic imaging, radiotherapy, fluoroscopically-guided interventional procedures, and nuclear medicine have contributed to the evolution and improvement of patient prognosis and medical care [2–4]. Such technological developments have led to an exponential increase in radiation use for medical purposes and hence in radiation exposure for professionals [5, 6].

Occupational cohorts are important for the study of health risks following protracted exposure to low doses of radiation, and medical workers were among the first to be the focus of such research [7]. It rapidly became apparent that exposure to ionizing radiation could lead to health risks for the personnel operating the equipment [8]. Short-term adverse clinical effects due to ionizing radiation use were reported among the pioneers of radiology soon after their use began, such as burn-like dermatitis, ulceration, reduced white blood cell count, or eye irradiation [9]. Epidemiological studies of medical radiation workers have also identified long-term excess risks of leukemia, skin, and female breast cancer in those employed before 1950 who were exposed to high doses of ionizing radiation [6]. This prompted to the emergence of professional societies and radiological protection committees who developed recommendations for medical workers, leading to the implementation of radiation protection measures such as maximum annual dose limits and personal protective equipment (e.g, leaded glass goggles, lead shielding for the operator) [10, 11]. A downward trend in average annual occupational exposure of medical radiation workers from around 70 mSv prior to 1939 down to around 2 mSv in the late 1970s and below 1 mSv today has been observed [8]. Although the exposure of medical workers has largely decreased over the years, medical uses of ionizing radiation have increased substantially over time and in new areas of medicine such as interventional cardiology, resulting in a need for new epidemiologic cohort studies representative of current practices. These studies will provide valuable insights on occupational radiation doses and risks of cancer and other diseases in these workers and will allow to assess whether current radiation protection regulations (annual dose limits, appropriate training, compliance with protective procedures, protective equipment, etc.) ensure adequate protection against the potential adverse effects of ionizing radiation.

Although epidemiological studies focusing on medical radiation workers have been carried out at the international level showing increased risk of occupational cataract [12] and lung cancer [13], none was available in France until now.

The ORICAMs (Occupational Radiation Induced CAncer in Medical staff) is a nationwide cohort of medical workers exposed to ionizing radiation in France. It is funded by the French national Institute for Radiological Protection and Nuclear Safety (IRSN) and was set up in 2011. The overall objective of the ORICAMs study is to investigate the risk of radiation-associated cancer and non-cancer mortality among medical staff exposed to ionizing radiation in their occupational activity. The cohort will form the basis for a continued epidemiological follow-up of the French medical radiation workers, allowing for assessment of long-term effects of radiation exposure. A second objective of the study is to spearhead an international effort to initiate collaborative studies of cancer risk after ionizing radiation exposure based on cohorts with contemporary exposure to increase statistical power of the dose-response analyses.

## Materials and methods

### Description of the cohort

The ORICAMs study is a retrospective cohort including medical workers exposed to ionizing radiation in the course of their work in France. Occupational exposure to ionizing radiation of workers in France is registered in SISERI, the national system for occupational dosimetry registration maintained by the IRSN, over their entire career. To be included in ORICAMs, workers had to have at least one dose recorded in SISERI (also including zero doses) between January 2002 and December 2012. The temporal criterion enables to overcome the limited data present in French registers before 2002. It also allows to collect data reflecting contemporary working exposures and conditions. Furthermore, workers were included if vital status and cause of death, if any, were recorded in national registers.

**Fig 1:**
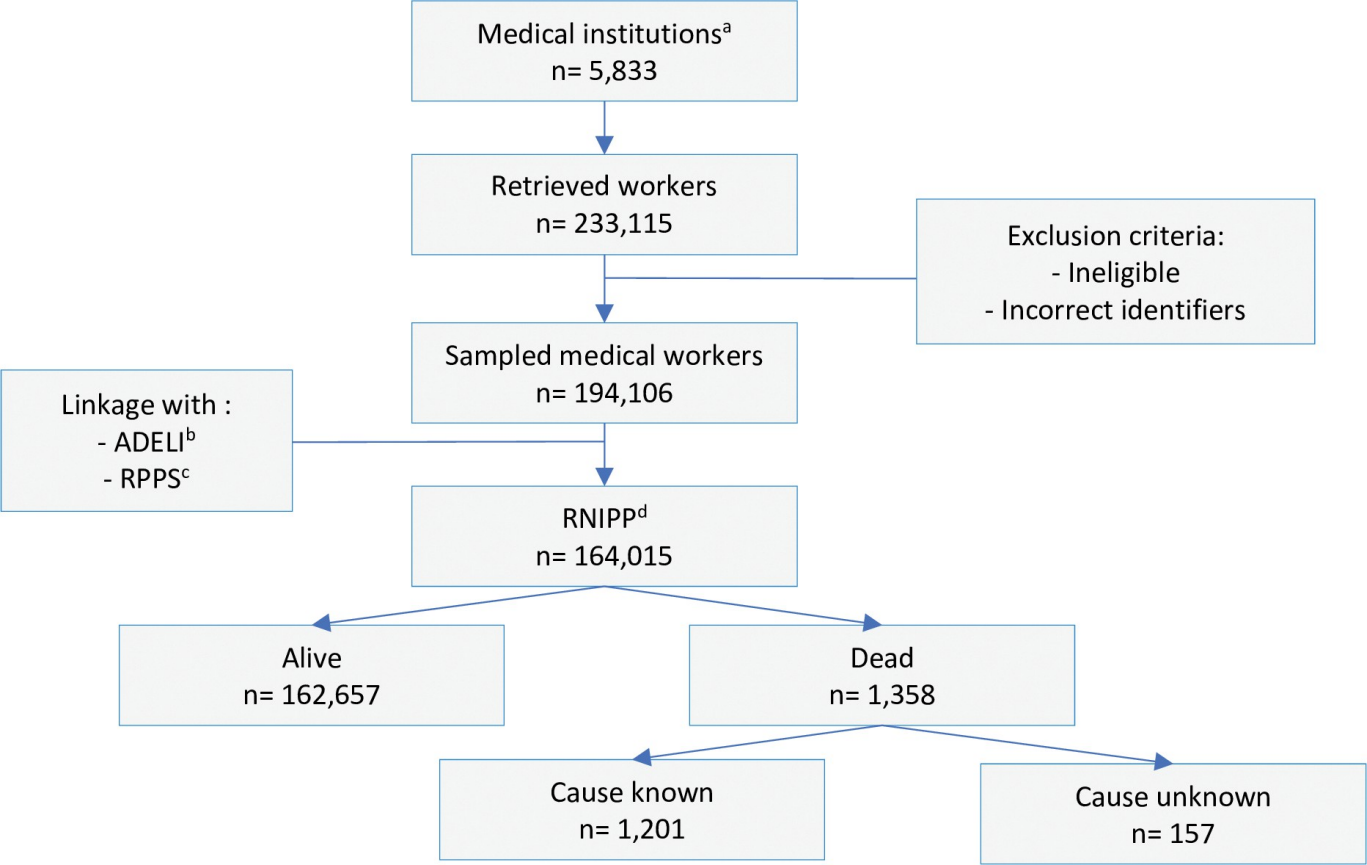
Flowchart of the ORICAMs cohort constitution. ^a^ obtained through linkage with the SISERI (Système d’Information de la Surveillance de l’Exposition aux Rayonnement Ionisants) database. ^b^ ADELI: Automatisation DEs Listes. ^c^ RPPS: Répertoire Partagé des Professionnels intervenant dans le système de Santé. ^d^ RNIPP: Répertoire National d’Identification des Personnes Physiques.

Fig 1 describes the cohort development process. Firstly, all the French medical institutions were extracted from the SISERI database according to their medical activity area between 2002 and 2012 (N = 5,833). Then all the medical staff working in these institutions and with a medical or a paramedical activity were selected (n = 233,115). Only workers with sufficient information available in the SISERI database were retained, e.g., name, sex, date of birth, and dosimetric information (n = 194,106). For each medical staff, retrieved information from the SISERI database were completed by linkage using French directories of healthcare professionals (RPPS and ADELI) providing details about professional activity. The RPPS provides information such as specialty of practice, work address, qualification, title, professional registration period, diploma, ministerial authorization specially for physicians, pharmacists, midwifes and dentists or dental surgeons. The ADELI database complies main identifying, administrative, and occupational information, especially for care assistants, and radiologic technologists.

### Vital status and outcome determination

The ORICAMs cohort is composed of 164,015 workers for whom the vital status is known. Workers were included if they were between 18 and 70 years of age at the start of the follow-up. For each worker, the follow-up started at the date of the first dosimetric record in the SISERI database or on Jan 1, 2002, whichever was later, and ended on Dec 31, 2013 or at the date of death if it occurred earlier. Vital statuses and medical causes of death were respectively obtained from the French National Register of Identification of Physical Persons (RNIPP, which is managed by the National Institute of Statistics and Economic Studies (ISEE) and compiles vital status of people born in France) and from the French National Institute of Health and Medical Research (CépiDC, which aims to collect every medical cause of death in France since 1968), respectively. Causes of death were coded according to the 10^th^ International Classification of Diseases (ICD-10) [14].

### Radiation exposure assessment

Medical workers are monthly or quarterly (according to their level of exposure) monitored by external passive dosimetry using Thermo Luminescence Dosimeters (TLD), Radio Photo Luminescence (RPL), or Optically Stimulated Luminescence (OSL) dosimeters worn at chest level under the lead apron, and considered as the whole body exposure. In addition, if there is a risk of exposure at the extremities (e.g., hand, wrist), extremity dosimetry is added to this passive whole-body dosimetry using TLD dosimeters. Dosimetric information or exposure measurements are regularly sent by the various data suppliers to the SISERI system *via* a secured internet access, using a strictly defined protocol and transmission formats. Individual dose equivalent Hp (10) is estimated from badges with a recording threshold of 0.05 mSv. Thus, Hp (10) and socio-professional information such as name, year of birth, profession, dates of first and last dosimetric recordings are available through the SISERI database. The dosimetric reconstruction for each worker is currently underway.

### Statistical analysis

We conducted external comparisons using standardized mortality ratios (SMRs) and 95% confidence intervals (CI) to compare the mortality in the cohort (observed number of deaths) with that in the French general population (expected number) [15] for all-causes and specific causes of death. The number of expected cases were calculated based on the data provided by the CépiDc. SMRs were standardized by age (1–24, 25–34, 35–44, 45–54, 55–64, 65–74 and 75–84), calendar year (2002–2006, 2006–2010 and 2010–2013), and gender (male and female). The expected number of deaths were obtained by multiplying the number of person-years in each gender-, age-, and calendar year-specific stratum using French mortality rates from 2002 to 2013.

SMR analyses were performed in R (R Foundation for Statistical Computing, Vienna, Austria) using the package popEpi. The statistical significance was defined by *p*< 0.05.

### Ethical approval

The study received ethical approval from the French national data protection commission (CNIL), deliberation N°DR-2012-567 of 3 December 2013. A general written information note concerning the implementation of the study has been disseminated *via* the occupational health departments of each health workers included in the study. Following this information note, workers could express their opposition to the research if desired. As this retrospective study was based on data already collected for other purposes, consent from the workers was not required. All data in this study was fully anonymized before we accessed them.

## Results and discussion

The main characteristics of the ORICAMs cohort are presented in Table 1.

**Table 1:** Description of the ORICAMs cohort.

Covariables	Male	Female	All
**Occupation** ^a^			
Nurse	7,838 (19.4)	32,625 (80.6)	40,463 (41.9)
Physician	21,836 (72.5)	8,280 (27.5)	30,116 (31.3)
Radiologic technologist	6,619 (30.7)	14,944 (69.3)	21,563 (22.4)
Others	2,116 (50.2)	2,096 (49.8)	4,212 (4.4)
**Calendar year of birth** ^a^			
≤ 1960	22,632 (53.4)	19,772 (46.6)	42,404 (25.8)
[1960–1980]	31,282 (37.1)	53,116 (62.9)	84,398 (51.5)
> 1980	11,568 (31.1)	25,645 (68.9)	37,213 (22.7)
**Follow-up**			
Person-years	562,943.2	819,512.9	1,382,456.1
Mean duration of follow-up ^b^	7.89 (3.5)	7.64 (3.5)	7.74 (3.5)
Mean age at cohort entry ^b^	37.36 (11.1)	33.16 (9.9)	34.84 (10.6)
Mean age at end of follow-up ^b^	45.87 (12.6)	41.39 (11.4)	43.18 (12.1)
**Duration of follow-up** ^a^			
≤ 1 year	3,364 (37.8)	5,548 (62.2)	8,912 (5.4)
[1–5 years]	15,570 (38.3)	25,064 (61.7)	40,634 (24.8)
[5–10 years]	16,983 (37.8)	27,991 (62.2)	44,974 (27.4)
>10 years	29,565 (42.5)	39,930 (57.5)	69,495 (42.4)
**Vital status** ^**a**^			
Alive	64,590 (39.7)	98,067 (60.3)	162,657 (99.2)
Dead	892 (65.7)	466 (34.3)	1,358 (0.8)
Unknown cause of death	110 (70.1)	47 (29.9)	157 (0.1)
**TOTAL**	65,482 (39.9)	98,533 (60.1)	164,015

^a^ N(%)

^b^ mean (SD); Others comprise: dental surgeon, pharmacist and midwife.

Among the 164,015 medical radiation workers included, there were more female (n = 98,533, 60%) than male (n = 65,482, 40%), and the majority of the workers were born after 1960 (74%). The cohort is composed mainly of nurses (42%), physicians (31%), and radiologic technologists (22%). Mean age was 34.8 (± 10.56) years at inclusion in the cohort, and 43.2 (± 12.07) years at the end of the follow-up. The cohort accrued a total of 1,382,456 person-years, resulting from an average follow-up of 8 years. At the end of the follow-up, 99% of the cohort were reported to be alive. A total of 1,358 deaths (892 cases in male and 466 in female) were recorded, including 532 (39%) from cancers, 395 (29%) from non-cancer diseases, 274 (20%) from external causes, and 157 (12%) causes of deaths were unknown.

SMRs by gender and for all the 164,015 medical workers are presented in Table 2.

**Table 2:** Standardized mortality ratios* (SMR), given for both gender and for all causes of death in the ORICAMs cohort.

	Male			Female			All		
**Causes of death (ICD-10)**	**O**	**E**	SMR* **(95% CI)**	**O**	**E**	SMR* **(95% CI)**	**O**	**E**	SMR* **(95% CI)**
**All known causes** (A00-Y89)	892	2,527.7	**0.35 (0.33, 0.38)**	466	1,132.5	**0.41 (0.38, 0.45)**	1,358	3,660.2	**0.37 (0.35, 0.39)**
**Cancer sites**									
All cancer combined (C00-C97)	301	977.8	**0.31 (0.27, 0.34)**	231	543.8	**0.43 (0.37, 0.48)**	532	1,521.6	**0.35 (0.32, 0.38)**
Lip, oral cavity and pharynx (C00-C14)	9	69.8	**0.13 (0.07, 0.24)**	2	10.8	**0.18 (0.05, 0.74)**	11	80.6	**0.14 (0.08, 0.25)**
Esophageal (C15)	7	47.2	**0.15 (0.07, 0.31)**	2	6.5	0.31 (0.08, 1.23)	9	53.7	**0.17 (0.09, 0.32)**
Stomach (C16)	7	30.9	**0.23 (0.11, 0.47)**	8	11.5	0.70 (0.35, 1.40)	15	42.4	**0.35 (0.21, 0.59)**
Colon, rectum, and anus (C18-C21)	30	71.3	**0.42 (0.29, 0.60)**	23	38.5	**0.60 (0.40, 0.90)**	53	109.8	**0.48 (0.37, 0.63)**
Liver (C22)	14	59.9	**0.23 (0.14, 0.39)**	7	10.8	0.65 (0.31, 1.36)	21	70.7	**0.30 (0.19, 0.46)**
Pancreas (C25)	36	50.1	**0.72 (0.52, 0.99)**	14	23.1	0.61 (0.36, 1.02)	50	73.2	**0.68 (0.52, 0.90)**
Larynx (C32)	3	37.7	**0.08 (0.03, 0.25)**	0	13.9	-	3	51.6	**0.06 (0.02, 0.18)**
Trachea, bronchi, and lung (C33-C34)	101	306.1	**0.33 (0.27, 0.40)**	49	123.4	**0.40 (0.30, 0.53)**	150	429.5	**0.35 (0.30, 0.41)**
Melanoma (C43)	8	12.9	0.62 (0.31, 1.23)	4	10.5	0.38 (0.14, 1.01)	12	23.4	**0.51 (0.29, 0.89)**
Breast (C50)	0	1.7	-	54	140.5	**0.38 (0.29, 0.50)**	54	142.2	**0.38 (0.29, 0.50)**
Uterus (C53)	-	-	-	5	16.2	**0.31 (0.10, 0.72)**	-	-	-
Ovary (C56)	-	-	-	14	30.7	**0.46 (0.25, 0.77)**	-	-	-
Prostate (C61)	10	27.5	**0.36 (0.17, 0.67)**	-	-	-	-	-	-
Kidney (C64)	7	21.2	**0.33 (0.16, 0.69)**	1	6.9	0.14 (0.02, 1.02)	8	28.1	**0.28 (0.14, 0.57)**
Bladder (C67)	6	24.4	**0.25 (0.11, 0.55)**	2	3.9	0.52 (0.13, 2.08)	8	28.3	**0.28 (0.14, 0.57)**
Brain and CNS (C70-C72)	17	29.9	**0.57 (0.35, 0.91)**	12	19.3	0.62 (0.35, 1.09)	29	49.2	**0.59 (0.41, 0.85)**
Thyroid (C73)	2	23.9	**0.08 (0.02, 0.33)**	0	15.8	-	2	39.7	**0.05 (0.01, 0.20)**
Hodgkin’s diseases and lymphoma (C81-C86)	6	23.9	**0.25 (0.11, 0.56)**	7	11.1	0.63 (0.30, 1.32)	13	35.0	**0.37 (0.22, 0.64)**
Leukemia (C91-C95)	9	12.5	0.72 (0.38, 1.39)	3	8.4	0.36 (0.11, 1.10)	12	20.9	0.57 (0.33, 1.01)
**Non-cancer**									
Certain infectious and parasitic diseases (A00-B99)	17	53.1	**0.32 (0.20, 0.52)**	3	23.6	**0.13 (0.04, 0.39)**	20	76.7	**0.26 (0.17, 0.40)**
Non-malignant tumors (D00-D48)	4	24.1	**0.17 (0.06, 0.44)**	3	13.1	**0.23 (0.07, 0.71)**	7	37.2	**0.19 (0.09, 0.39)**
Diseases of the blood (D50-D89)	3	6.9	0.43 (0.14, 1.34)	0	4.7	**-**	3	11.6	**0.26 (0.08, 0.79)**
Endocrine, nutritional, and metabolic diseases (E00-E89)	12	55.5	**0.22 (0.12, 0.38)**	2	27.1	**0.07 (0.02, 0.30)**	14	82.6	**0.17 (0.10, 0.29)**
Mental and behavioral disorders (F01-F99)	14	89.9	**0.16 (0.09, 0.26)**	6	27.8	**0.22 (0.10, 0.48)**	20	117.7	**0.17 (0.11, 0.26)**
Due to use of alcohol (F10)	8	63.8	**0.13 (0.06, 0.25)**	2	15.4	**0.13 (0.03, 0.52)**	10	79.2	**0.13 (0.07, 0.23)**
Diseases of the nervous system and sense organs (G00-H95)	20	66.9	**0.29 (0.19, 0.46)**	6	40.2	**0.15 (0.07, 0.33)**	26	107.1	**0.24 (0.17, 0.36)**
Circulatory system diseases (I00-I99)	104	400.5	**0.26 (0.21, 0.31)**	23	126.2	**0.18 (0.12, 0.27)**	127	526.7	**0.24 (0.20, 0.29)**
Ischaemic heart diseases (I20-I25)	44	159.7	**0.28 (0.21, 0.37)**	1	26.5	**0.04 (0.01, 0.27)**	45	186.2	**0.24 (0.18, 0.32)**
Cerebrovascular diseases (I60-I69)	23	71.7	**0.32 (0.21, 0.48)**	9	37.1	**0.24 (0.13, 0.47)**	32	108.8	**0.29 (0.21, 0.42)**
Diseases of the respiratory system (J00-J99)	15	71.8	**0.21 (0.13, 0.35)**	3	25.9	**0.12 (0.04, 0.36)**	18	97.7	**0.18 (0.12, 0.29)**
Diseases of the digestive system (K00-K93)	26	168.4	**0.15 (0.11, 0.23)**	12	62.4	**0.19 (0.11, 0.34)**	38	230.8	**0.16 (0.12, 0.23)**
External causes (V01-Y89)	182	383.9	**0.47 (0.41, 0.55)**	92	154.8	**0.59 (0.48, 0.73)**	274	538.7	**0.51 (0.45, 0.57)**
Transport accidents (V01-V99, Y85)	25	73.7	**0.34 (0.23, 0.50)**	17	23.1	0.74 (0.46, 1.19)	42	96.8	**0.43 (0.32, 0.59)**
Accidental poisoning by and exposure to noxious substances (X40-X49)	10	12.9	0.78 (0.42, 1.44)	9	7.8	1.15 (0.59, 2.21)	19	20.7	0.91 (0.58, 1.44)
Intentional self-harm (X60-X84, Y87.0)	116	176.2	**0.66 (0.55, 0.79)**	56	76.7	**0.73 (0.56, 0.95)**	172	252.9	**0.68 (0.59, 0.79)**

CNS: Central Nervous System; SMR: Standardized Mortality Ratio; O: Observed; E: Expected.

* SMRs were standardized by age (1–24, 25–34, 35–44, 45–54, 55–64, 65–74 and 75–84) and calendar year (2002–2006, 2006–2010 and 2010–2013)

Bold values are significant SMRs.

For all causes of deaths, a significant lower mortality for both male (SMR = 0.35; 95% CI 0.33, 0.38; n_deaths_ = 892) and female (SMR = 0.41; 95% CI 0.38, 0.45; n_deaths_ = 466) compared with national rates were observed. Similarly, significant decreases were found for all malignant neoplasms (SMR = 0.35; 95% CI 0.32, 0.38; n_deaths_ = 532; for both genders together) and each specific cancer type, except for leukemia (SMR = 0.57; 95% CI 0.33, 1.01; n_deaths_ = 12), cause for which mortality was also lower than in the general population, but not significantly so because the expected and observed numbers of deaths were relatively small and close. Concerning other causes of death, significant lower mortality than in the general population was observed for most of the studied causes, such as mental and behavioral disorders (SMR = 0.17; 95% CI 0.11, 0.26; n_deaths_ = 20) and circulatory diseases (SMR = 0.24; 95% CI 0.20, 0.29; n_deaths_ = 127). As a comparison, studies in other countries reported similar results, i.e. decreased all-cancer mortality compared with national rates: SMR = 0.64 (95% CI: 0.62, 0.66), SMR = 0.61 (95% CI: 0.55, 0.67), and SMR = 0.57 (95% CI: 0.52, 0.63), for the American [13], Korean [22] and Canadian [23] medical radiation workers cohorts, respectively.

Results of the gender-specific cancer and non-cancer mortality analyses showed the same pattern (SMRs significantly lower than 1 for almost all specific causes of death). However, some results were limited among female due to low numbers of deaths for specific causes of death.

Subgroup analyses for all causes of death (S1 Table in S1 File) and all combined cancer causes of death (S2 Table in S1 File) by job title, year of entry into the cohort, and year of birth did not show any particular pattern.

In the future when individual dose assessment will be completed, dose-response analysis will be performed. We plan to use a nested case-control study design to be able to collect additional information on lifestyle factors in occupational medical records for cases and controls. For this purpose, all professionals who died of a disease of interest in the cohort will be included as cases in the case-control study and will be randomly matched with 5 controls on sex, year of birth, and date of cohort entry. Occupational exposure to ionizing radiation will be provided by SISERI, and additional information on potential risk factors will be provided by worker’s occupational medical records (e.g., weight, type of work, smoking status, alcohol intake, predisposition, etc).

The strengths of this study include the large sample size of the cohort involving medical workers exposed to ionizing radiation between 2002 and 2012 in France, a design conducted at the national level, and the use of national registries that ensure accurate and unbiased data collection. This is the first study conducted in France about medical radiation workers occupationally exposed, allowing a basis for continued follow-up. The inclusion criterion that medical workers were included in the cohort if they were exposed between 2002 and 2012 allows for a reflection of current ionizing radiation-exposures and working conditions. The large size of the cohort offers the possibility to include gender-specific analyses, to study rare diseases, and to investigate the link between ionizing radiation-exposure and various diseases, including in female, a population rarely studied in radiation occupational cohorts. Indeed, the present cohort is mainly composed of female (60%), which differs from previous large cohorts of ionizing radiation-exposed workers (e.g. nuclear workers, uranium miners, pilots, etc) in which they are poorly represented (0 to 30% of female) [16–19], while sensitivity to ionizing radiation-exposure may vary by sex [20].

Furthermore, this cohort will be part of the international BECOME (Brain cancEr risk in joint COhort of MEdical workers) project, aiming to carry out pooled analysis of three nested case-control studies from three national cohorts of ionizing radiation-exposed medical workers in France, Korea and the United States. The aim of the collaborative project is to quantify the radiation-induced risk of death from central nervous system tumor in relation to occupational doses from records of exposed medical radiation workers.

However, in the present study, the relatively short follow-up period (8 years of follow-up on average) and the young age at inclusion and at the end of the follow-up leads to a weak number of deaths (0.8% of medical workers were recorded as deceased at the end of the follow-up, while nearly 89% were under the age of 60), which may limit our interpretations. Continuing the follow-up will improve the statistical power of analyses as the number of deaths will increase.

Another weakness of the study is the expected healthy worker effect (HWE) [21] we observed. The results showed significantly lower mortality in the cohort than in the general population, likely reflecting the HWE, i.e. a better health status of workers and also closer monitoring of their health by occupational health services. The HWE is reported in many occupational studies, like in the American [13], Korean [22] and Canadian [23] medical radiation workers cohorts that also reported decreased all-cancer mortality compared with national rates. The influence of the HWE varies between occupational cohorts (e.g. medical workers vs nuclear industry workers) [24] but is likely to be more pronounced in medical radiation workers as they have better access to medical care than others and may have healthier lifestyle (e.g. nonsmoker, healthy diet, exercise). Furthermore, due to the absence of a national cancer registry in France, we were unable to study disease incidence, thus precluding the study of low mortality diseases rates such as thyroid or breast cancer. The absence of lifestyle factors are important limitations, even if job type can be considered as a proxy of the socioeconomic status. However, we plan to use a nested case control methodology in our future analyses in order to be able to retrieve from occupational medical records information on lifestyle and other risk factors for the studied outcomes.

## Conclusion

This study is the first analysis of the ORICAMs cohort of medical radiation workers and showed a significantly lower mortality compared to the general population, which may reflect the HWE. The individual ionizing radiation dose available for each worker were not used in this study. Since it is not feasible to use dosimetric data directly from the databases as it is necessary to take into account the detection threshold, the periodicity, and whether the exposure has changed over the years, the analysis of the dose for each individual is currently underway. Nevertheless, in future analyses of the cohort, the dose-response analysis will be assessed, as in the international project.

## Supporting information

S1 File Supplementary data are available at online. (DOCX)
