# Supplementary material for: Cohort profile: ORICAMs, a French cohort of medical workers exposed to low-dose ionizing radiation
Source: PLoS One. 2023 Jun 8;18(6):e0286910. doi: 10.1371/journal.pone.0286910 (PMC10249798; doi:10.1371/journal.pone.0286910)
Supplement: S1 File — (DOCX) [file pone.0286910.s001.docx]

**Cohort Profile: ORICAMs, a French cohort of medical workers exposed to low-dose ionizing radiation**

*Julie Lopes^1*^, Clémence Baudin^1^, Juliette Feuardent^2^, Hervé Roy^2^, Sylvaine Caër-Lorho^1^, Klervi Leuraud^1^, Marie-Odile Bernier^1^*

*^1^Laboratory of epidemiology (LEPID) – Institute for Radiological Protection and Nuclear Safety (IRSN), 92262 Fontenay-aux-Roses, France.*

*^2^Office for the analysis and monitoring of occupational exposure (BASEP) – Institute for Radiological Protection and Nuclear Safety (IRSN), 92262 Fontenay-aux-Roses, France.*

## Supplementary data

Table S1: Standardized mortality ratios (SMRs) by gender for all causes of death according to the profession, the year of entry into the cohort, and the age of entry into the cohort.

|  | **Male** | | | **Female** | | | **All** | | |
| --- | --- | --- | --- | --- | --- | --- | --- | --- | --- |
|  | **O** | **E** | **SMR (95% CI)** | **O** | **E** | **SMR (95% CI)** | **O** | **E** | **SMR (95% CI)** |
| **Occupation** |  |  |  |  |  |  |  |  |  |
| Nurse | 60 | 204.0 | 0.29 (0.23, 0.38) | 115 | 326.8 | 0.35 (0.29, 0.42) | 175 | 530.8 | 0.33 (0.28, 0.38) |
| Radiologist technologist | 88 | 242.3 | 0.36 (0.29, 0.45) | 63 | 184.5 | 0.34 (0.27, 0.44) | 151 | 426.8 | 0.35 (0.30, 0.42) |
| Physician | 4 | 1,038.7 | 0.00 (0.00, 0.01) | 2 | 104.7 | 0.02 (0.00, 0.08) | 6 | 1,143.4 | 0.01 (0.00, 0.01) |
| Others | 0 | 50.9 | - | 1 | 12.5 | 0.08 (0.01, 0.57) | 1 | 63.4 | 0.02 (0.00, 0.09) |
| **Year of entry into the cohort** |  |  |  |  |  |  |  |  |  |
| ≤ 2005 | 776 | 2,228.7 | 0.35 (0.32, 0.37) | 400 | 988.5 | 0.40 (0.37, 0.45) | 1,176 | 3,217.2 | 0.37 (0.35, 0.39) |
| ]2005 – 2010] | 103 | 267.7 | 0.38 (0.32, 0.47) | 62 | 129.3 | 0.48 (0.37, 0.62) | 165 | 397.0 | 0.42 (0.36, 0.48) |
| > 2010 | 13 | 31.2 | 0.42 (0.24, 0.72) | 4 | 14.7 | 0.27 (0.10, 0.73) | 17 | 45.9 | 0.37 (0.23, 0.59) |
| **Age (years) of entry into the cohort** |  |  |  |  |  |  |  |  |  |
| ≤ 24 | 18 | 41.6 | 0.43 (0.27, 0.69) | 17 | 41.2 | 0.41 (0.26, 0.66) | 35 | 82.8 | 0.42 (0.29, 0.59) |
| ] 25 – 34] | 76 | 226.3 | 0.34 (0.27, 0.42) | 85 | 184.0 | 0.46 (0.37, 0.57) | 161 | 410.3 | 0.39 (0.33, 0.46) |
| ] 35 – 44] | 185 | 604.8 | 0.31 (0.26, 0.35) | 110 | 366.4 | 0.30 (0.25, 0.36) | 295 | 971.2 | 0.30 (0.27, 0.34) |
| ] 45 – 54] | 369 | 1,108.4 | 0.33 (0.30, 0.37) | 195 | 421.8 | 0.46 (0.40, 0.53) | 564 | 1,530.2 | 0.37 (0.34, 0.40) |
| ] 55 – 64] | 184 | 417.9 | 0.44 (0.38, 0.51) | 45 | 85.6 | 0.53 (0.39, 0.70) | 229 | 503.5 | 0.45 (0.40, 0.52) |
| ] 65 – 74] | 4 | 6.7 | 0.60 (0.22, 1.59) | 1 | 0.8 | 1.22 (0.17, 8.65) | 5 | 7.5 | 0.67 (0.22, 1.55) |

*Others comprise: dental surgeon, pharmacist and midwife.*

Table S2: Standardized mortality ratios (SMRs) by gender for all combined cancer causes of death according to the profession, the year of entry into the cohort, and the age of entry into the cohort.

|  | **Male** | | | **Female** | | | **All** | | |
| --- | --- | --- | --- | --- | --- | --- | --- | --- | --- |
|  | **O** | **E** | **SMR (95% CI)** | **O** | **E** | **SMR (95% CI)** | **O** | **E** | **SMR (95% CI)** |
| **Occupation** |  |  |  |  |  |  |  |  |  |
| Nurse | 13 | 66.3 | 0.20 (0.11, 0.34) | 61 | 149.6 | 0.41 (0.32, 0.52) | 74 | 215.9 | 0.34 (0.27, 0.43) |
| Radiologist technologist | 32 | 89.3 | 0.36 (0.25, 0.51) | 30 | 86.6 | 0.35 (0.24, 0.50) | 62 | 175.9 | 0.35 (0.27, 0.45) |
| Physician | 1 | 416.5 | 0.00 (0.00, 0.02) | 1 | 51.3 | 0.02 (0.00, 0.14) | 2 | 467.8 | 0.00 (0.00, 0.02) |
| Other | 0 | - | - | 0 | - | - | 0 | - | - |
| **Year of entry into the cohort** |  |  |  |  |  |  |  |  |  |
| ≤ 2005 | 272 | 876.9 | 0.31 (0.28, 0.35) | 202 | 482.9 | 0.42 (0.36, 0.48) | 474 | 1,359.8 | 0.35 (0.32, 0.38) |
| ]2005 – 2010] | 28 | 90.8 | 0.31 (0.21, 0.45) | 29 | 54.9 | 0.53 (0.37, 0.76) | 57 | 145.7 | 0.39 (0.30, 0.51) |
| > 2010 | 1 | 9.9 | 0.10 (0.01, 0.71) | 0 | 6.0 | - | 1 | 15.9 | 0.06 (0.00, 0.35) |
| **Age (years) of entry into the cohort** |  |  |  |  |  |  |  |  |  |
| ≤ 24 | 2 | 3.9 | 0.50 (0.13, 2.01) | 4 | 10.0 | 0.40 (0.15, 1.06) | 6 | 13.9 | 0.43 (0.16, 0.93) |
| ] 25 – 34] | 4 | 33.5 | 0.12 (0.04, 0.32) | 33 | 63.4 | 0.52 (0.37, 0.73) | 37 | 96.9 | 0.38 (0.27, 0.53) |
| ] 35 – 44] | 41 | 195.5 | 0.21 (0.15, 0.28) | 45 | 171.9 | 0.26 (0.20, 0.35) | 86 | 367.4 | 0.23 (0.19, 0.29) |
| ] 45 – 54] | 134 | 490.5 | 0.27 (0.23, 0.32) | 109 | 228.3 | 0.48 (0.40, 0.58) | 243 | 718.8 | 0.34 (0.30, 0.38) |
| ] 55 – 64] | 91 | 194.5 | 0.47 (0.38, 0.57) | 29 | 49.4 | 0.59 (0.41, 0.84) | 120 | 243.9 | 0.49 (0.41, 0.59) |
| ] 65 – 74] | 3 | 2.9 | 1.05 (0.34, 3.26) | 1 | 0.4 | 2.75 (0.39, 19.52) | 4 | 3.3 | 1.24 (0.34, 3.18) |

*Others comprise: dental surgeon, pharmacist and midwife.*
